# Prevalence of chronic medical conditions in Switzerland: exploring estimates validity by comparing complementary data sources

**DOI:** 10.1186/1471-2458-14-1157

**Published:** 2014-11-07

**Authors:** Ueli Zellweger, Matthias Bopp, Barbara M Holzer, Sima Djalali, Vladimir Kaplan

**Affiliations:** Epidemiology, Biostatistics and Prevention Institute, University of Zurich, Hirschengraben 84, Zürich, CH-8001 Switzerland; Division of Internal Medicine, University Hospital Zurich, Zurich, Switzerland; Institute of General Practice, University of Zurich, Zurich, Switzerland; District Hospital Freiamt Muri, Muri, Switzerland; Members of the FIRE Study Group, c/o Institute of General Practice, University of Zurich, Zurich, Switzerland; Center of Competence Multimorbidity, University of Zurich, Zurich, Switzerland

**Keywords:** Chronic medical condition, Prevalence, Population, Hypertension, Diabetes mellitus, Dyslipidemia, Obesity

## Abstract

**Background:**

Prevalence estimates of chronic medical conditions and their multiples (multimorbidity) in the general population are scarce and often rather speculative in Switzerland. Using complementary data sources, we assessed estimates validity of population-based prevalence rates of four common chronic medical conditions with high impact on cardiovascular health (diabetes mellitus, hypertension, dyslipidemia, obesity).

**Methods:**

We restricted our analyses to patients 15-94 years old living in the German speaking part of Switzerland. Data sources were: Swiss Health Survey (SHS, 2007, n = 13,580); Family Medicine ICPC Research using Electronic Medical Record Database (FIRE, 2010-12, n = 99,441); and hospital discharge statistics (MEDSTAT, 2009-10, n = 883,936). We defined chronic medical conditions based on use of drugs, diagnoses, and measurements.

**Results:**

After a careful harmonization of the definitions, a high degree of concordance, especially regarding the age- and gender-specific distribution patterns, was found for diabetes mellitus (defined as drug use or diagnosis in SHS, drug use or diagnosis or blood glucose measurement in FIRE, and ICD-10 codes E10-14 as secondary diagnosis in MEDSTAT) and for hypertension (defined as drug use alone in SHS and FIRE, and ICD-10 codes I10-15 or I67.4 as secondary diagnosis in MEDSTAT). A lesser degree of concordance was found for dyslipidemia (defined as drug use alone in SHS and FIRE, and ICD-10 code E78 in MEDSTAT), and for obesity (defined as BMI ≥ 30 kg/m2 derived from self-reported height and weight in SHS, from measured height and weight or diagnosis of obesity in FIRE, and ICD-10 code E66 as secondary diagnosis in MEDSTAT). MEDSTAT performed well for clearly defined diagnoses (diabetes, hypertension), but underrepresented systematically more symptomatic conditions (dyslipidemia, obesity).

**Conclusion:**

Complementary data sources can provide different prevalence estimates of chronic medical conditions in the general population. However, common age and sex patterns indicate that a careful harmonization of the definition of each chronic medical condition permits a high degree of concordance.

## Background

Due to demographic ageing and advances in medical care the prevalence of chronic medical conditions and multimorbidity (defined as more than one chronic medical condition in one individual [1]) is increasing worldwide. Estimates from the United States suggest that by 2020, nearly 50% of the population will have at least one chronic medical condition, with most suffering from multimorbidity [2].

However, measuring prevalence rates of chronic medical conditions poses challenges, because of varying case definitions (medical nosology, inclusion of symptoms, laboratory values, and prescribed medication), different methods of case identification (self-report, clinical exam, registry), and diverse sampling strategies (general population, general practice population, population in specific medical care settings). Population surveys using solely information on self-reported diagnoses are additionally influenced by informational and recall biases. Therefore, prevalence estimates vary widely across different studies.

Despite its significance for public health, population based estimates of prevalence rates of chronic medical conditions and multimorbidity are scarce in Switzerland. A recently published study explored the prevalence of chronic medical conditions and multimorbidity in primary care in the German speaking part of Switzerland and found that multimorbidity was more common than the most prevalent single chronic medical condition, hypertension (15% vs. 9%) [3]. However, a relative small number of participating primary care providers, varying coding practices, and a potential selection bias (individuals consulting a doctor might be sicker than the average population), did not allow an uncritical generalization of the results to the entire population.

“Cross-validation” of different health statistics has been shown to be a promising way to obtain valid estimates of prevalence rates of diabetes mellitus in Switzerland [4]. Even when absolute numbers diverge, similarities regarding age- and gender-specific distribution patterns increase the validity of the estimates. However, simply contrasting data from primary care with survey data will not suffice, as has been shown for prevalence estimates of multimorbidity in Canadian settings [5].

Therefore, we explored similarities and differences between Swiss health statistics (Swiss Health Survey [SHS], primary care data [FIRE], and hospital discharge statistics [MEDSTAT]), regarding four common chronic conditions with high impact on cardiovascular health: hypertension, diabetes mellitus, dyslipidemia, and obesity. We hypothesized that with a careful definition of each chronic condition and each data source we will be able to harmonize the estimates despite different sampling techniques (self-report, physician report, drug prescription, measurement). We also expected to find hints concerning over- or under-reporting of specific chronic medical conditions in specific data sources.

## Methods

### Data sources

#### Swiss health survey 2007 (SHS)

The Swiss Health Survey (SHS) is conducted since 1992 every five years, targets the general population of Switzerland ≥15 years, and provides nationally representative information on health-related behavior and attitudes, as well as frequency and type of healthcare utilization. Eligible subjects are chosen by stratified random sampling (based on telephone registry) of private households with landline telephone. Within each contacted household, one member is randomly selected (random-random-procedure) for computer assisted telephone interview. Details regarding the sampling procedure are provided elsewhere (http://www.bfs.admin.ch/bfs/portal/de/index/infothek/erhebungen__quellen/blank/blank/ess/04.html). In 2007, 17,931 individuals participated in the interview (participation rate 66%).

#### Primary care data 2010-2012 (FIRE)

We obtained primary care based data from the Swiss Family Medicine International Classification of Primary Care (ICPC) Research using Electronic Medical Record project (FIRE), which was initiated 2009 by the SGAM (Association of Swiss General Practitioners) and coordinated by the Institute of General Practice at the University of Zurich, Zurich, Switzerland [6]. Primary care physicians in the German speaking part of Switzerland (who used electronic patient records) provided voluntarily standardized, anonymized data on all patient-physician encounters (patient’s demographics, vital signs, diagnostic codes using the second version of the International Classification of Primary Care [ICPC-2], laboratory values, and medication using the Anatomical Therapeutic Chemical Classification System [ATC]). Between May 2010 and April 2012, there were 556,353 consultations in 113,318 patients. For a valid comparison of MEDSTAT (ICD-10 classification system) and FIRE (ICPC-2 classification system), we used an IT-tool programmed by the Academic Medical Center, University Amsterdam, that translates ICPC-2 to ICD-10 codes [7].

#### Hospital discharge statistics 2009-2010 (MEDSTAT)

In Switzerland, hospital discharges are routinely registered since 1998. The data include gender, age, and region of residence, other administrative variables, and one principal diagnosis and up to 49 additional (“secondary”) diagnoses encoded according to the International Statistical Classification of Diseases (ICD-10). Hospitalizations concerning the same individual can be identified (http://www.bfs.admin.ch/bfs/portal/de/index/infothek/erhebungen__quellen/blank/blank/mkh/01.html). In 2009 and 2010, MEDSTAT constituted of 2.673 million hospital discharges in 1.715 million patients.

### Data protection

SHS and MEDSTAT are administered by the Swiss Federal Statistics Office as a part of its legal mission. The use of fully anonymized individual data from these sources is subject to specific data contracts with the Institute of Social and Preventive Medicine. FIRE data are fully anonymized and stored on a central server. Only two of the authors (SD, VK) had access to the data. According to the current Swiss law on human research (Humanforschungsgesetz, HFG) retrospective analyses of anonymized medical routine data do not requires approval by the regional ethics committee http://www.bag.admin.ch/themen/medizin/00701/00702/07558/.

### Definitions of chronic conditions

#### Health survey 2007 (SHS)

We used the following items of the survey to define the chronic conditions (Table 1):

**Table 1:** **Case definition of diabetes mellitus, hypertension, dyslipidemia, and obesity for different data sources**

	Health survey 2007 (SHS)	Primary care 2010-2012 (FIRE*)	Hospital discharge statistics 2009-2010 (MEDSTAT ^†^)
**Diabetes mellitus**	**Drug use**	**Drug use**	
	Did you take medication for diabetes or injected insulin in the last seven days?	Drugs used in diabetes (A10)	
	**Diagnosis**	**Diagnoses**	**Diagnosis**
	Were you ever told by a physician to have diabetes?	Diabetes insulin dependent (T89)	Diabetes mellitus (E10-E14)
		Diabetes non-insulin dependent (T90)	
		**Measurement**	
		HbA1c ≥6.5% or random plasma	
		glucose ≥11.1 mmol/L	
**Hypertension**	**Drug use**	**Drug use**	
	Did you take any medication for high blood pressure in the last seven days?	Diuretics (C03)	
		Peripheral vasodilators (C04)	
		Beta blocking agents (C07)	
		Calcium channel blockers (C08)	
		Agents acting on renin-angiotensin (C09)	
	**Diagnosis**	**Diagnosis**	**Diagnosis**
	Were you ever told by a physician or another health professional to have high blood pressure?	Hypertension uncomplicated (K86)	Hypertension (I10-I15)
		Hypertension complicated (K87)	
		**Measurement**	
		Systolic blood pressure ≥140 mmHg or diastolic blood pressure ≥90 on two or more occasions	
**Dyslipidemia**	**Drug use**	**Drug use**	
	Did you take medication for high blood cholesterol in the last seven days?	Lipid modifying agents (C10)	
	**Diagnosis**	**Diagnosis**	**Diagnosis**
	Were you ever told by a physician or another health professional to have high blood cholesterol?	Lipid disorder (T93)	Disorders of lipid metabolism (E78)
		**Measurement**	
		Total cholesterol >5.17 mmol/L or triglycerides >1.69 mmol/L	
**Obesity**		**Diagnosis**	**Diagnosis**
		Obesity (T82)	Obesity (E66)
	**Measurement (reported)**	**Measurement**	
	BMI ≥30 kg/m2 derived from self-reported height and weight	BMI ≥30 kg/m2 derived from measured height and weight	

*International classification of primary care version 2.

^†^International statistical classification of diseases and related health problems, 10^th^ revision.

Diabetes mellitus: Did you take any medication for diabetes or used insulin in the last seven days? Were you ever told by a physician that you have diabetes? Hypertension: Did you take any medication for high blood pressure in the last seven days? Were you ever told by a physician or another health professional to have high blood pressure? Dyslipidemia: Did you take any medication for high cholesterol (lipids) in the last seven days? Were you ever told by your physician or another health professional to have high blood cholesterol (lipids)? Obesity: Body mass index (BMI) ≥30 kg/m2, derived from self-reported height and weight.

#### Primary care data 2010-2012 (FIRE)

We used disease-specific drugs, diagnostic codes, or laboratory values to define four chronic medical conditions (Table 1): Diabetes mellitus: Drugs used in diabetes mellitus (ATC codes A10); diagnostic codes for diabetes (ICPC-2 T89 and T90); either HbA1c ≥6.5% or random plasma glucose ≥11.1 mmol/L. Hypertension: Drugs used for high blood pressure (ATC codes C03, C04, C07, C08, C09); diagnostic codes for hypertension (ICPC-2 diagnoses K86 and K87); blood pressure, either systolic blood pressure ≥140 mmHg or diastolic blood pressure ≥90 mmHg on two or more occasions. Dyslipidemia: Drugs used as lipid modifying agents (ATC code C10); diagnostic code (ICPC-2 diagnosis T93; cholesterol or triglyceride (either total cholesterol ≥5.17 mmol/L or triglycerides ≥1.69 mmol/L. Obesity: BMI ≥30 kg/m2 derived from measured height and weight; diagnostic code for obesity (ICPC-2 T82). Information on measured height and weight was available for a subgroup of 26 primary care physicians who provided these measurements in at least 20% of patient encounters.

#### Hospital discharge statistics 2009-2010 (MEDSTAT)

We used all disease-specific ICD-10 codes to identify diabetes mellitus (E10-14), hypertension (I10-I15), lipid disorder (E78), and obesity (E66). To estimate the prevalence rates in the general population, we restricted our sample to patients hospitalized due to other conditions than those explored, because we assumed that patients with these chronic conditions as principal diagnosis have a substantially increased risk of hospital admission and would therefore be overrepresented compared to the general population.

### Analyses

We compared the age- and gender-specific prevalence rates for each definition of the four chronic medical conditions and for each data source. Because all FIRE primary care physicians were located in the German speaking part of Switzerland, we restricted all analyses to residents of that area, thus avoiding bias due to different operating customs as well as cultural and semantic disparities. Since SHS provides only information on individuals 15 years and older, we excluded individuals aged less than 15 years at the last consultation/hospitalization in FIRE (N = 7,287) and MEDSTAT (N = 89,135). Those aged >95 years were also excluded because of small absolute numbers (N = 7 for SHS 2007, N = 395 for FIRE 2010-12, N = 4,296 for MEDSTAT). Based on these selection criteria, the study population amounted to 13,580 for SHS 2007, 99,441 for FIRE 2010-12, and 883,936 for MEDSTAT 2009-10.

Generally, missing values, e.g. for diagnoses, could not be discerned from negative answers and had therefore to be handled as negation. Except for BMI in FIRE, missing values were extremely rare (N = 184 for BMI and N = 18 for all questions concerning diabetes in the SHS 2007, N = 7 for age or sex in FIRE 2010-12).

Overall rates were age-standardized using the WHO standard population “Europe”. We managed data and conducted analyses using SPSS® Version 18 and 19 (SPSS Inc., Chicago, IL, USA) and Stata® Version 11.2 (Stata Corporation, College Station, TX, USA; http://www.stata.com).

## Results

### Diabetes mellitus

**Figure 1:**
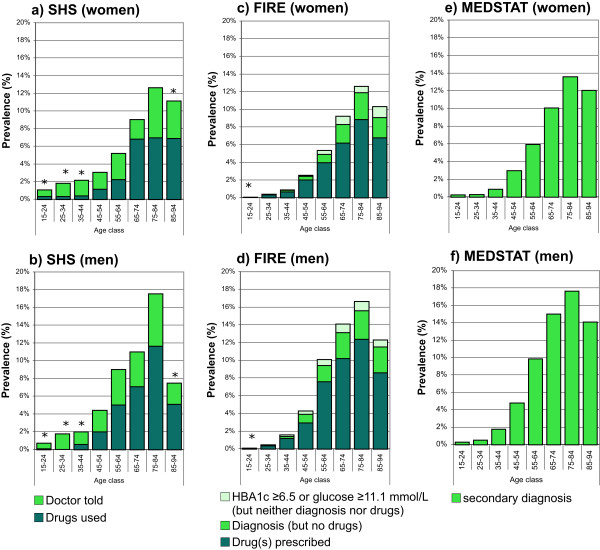
**Age- and gender-specific prevalence estimates of diabetes mellitus based on complementary data sources and various definitions.** For health survey (SHS) **(a** **and** **b)**, we defined cases as drug use alone, and drug use or diagnosis of diabetes. For primary care data (FIRE) **(c** **and** **d)**, we defined cases as drug use alone, drug use or diagnosis, and drug use or diagnosis or serum glucose measurement (serum glucose ≥11.1 mmol/L or HbA1c ≥6.5%). For hospital discharge statistics (MEDSTAT) **(e** **and** **f)**, we defined cases as ICD-10 codes E10-14 as secondary diagnoses. Results based on less than 30 observations are marked by an asterisk. (Data sources: Swiss Federal Statistical Office for Swiss Health Survey [SHS] and Hospital Discharge Statistics [MEDSTAT]; Swiss Family Medicine International Classification of Primary Care Research using Electronic Medical Record project for primary care data [FIRE]).

SHS: The standardized prevalence estimates of diabetes mellitus defined as self-reported drug use alone, and self-reported drug use or self-reported diagnosis of diabetes mellitus, were 1.7% (95% CI 1.4-1.9%) and 3.6% (95% CI 3.2-4.0%), and 2.3% (95% CI 2.0-2.7%) and 4.6% (95% CI 4.1-5.1%) for women and men, respectively. The age- and gender-specific prevalence rates of diabetes mellitus for the two different case definitions are provided in Figure 1a and b.FIRE: The standardized prevalence estimates of diabetes mellitus defined as drug prescription, drug prescription or diagnosis, drug prescription or diagnosis or measurement (serum glucose ≥11.1 mmol/L or HbA1c ≥6.5%), were 2.1% (95% CI 1.9-2.2%), 2.7% (95% CI 2.5-2.8%), 2.9% (95% CI 2.7-3.0%), and 3.4% (95% CI 3.2-3.5%), 4.3% (95% CI 4.1-4.5%), and 4.6% (95% CI 4.4-4.8%) for women and men, respectively. The age- and gender-specific prevalence rates of diabetes mellitus for the three different case definitions are provided in Figure 1c and d.MEDSTAT: The standardized prevalence rates of diabetes mellitus defined as ICD-10 codes E10-14 as secondary diagnoses were 3.2% (95% CI 3.2-3.2%), and 4.9% (95% CI 4.8-4.9%) for women and men, respectively. The age- and gender-specific prevalence rates of diabetes mellitus defined as ICD-10 codes E10-14 as secondary diagnoses are provided in Figure 1e and f.

### Hypertension

**Figure 2:**
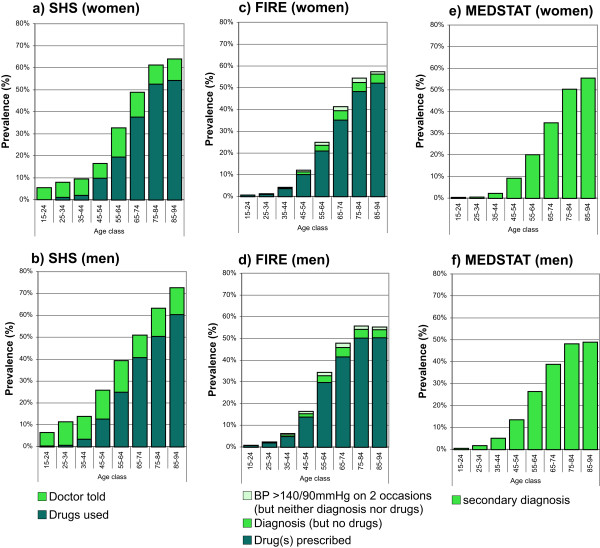
**Age- and gender-specific prevalence estimates of hypertension based on complementary data sources and various definitions.** For health survey (SHS) **(a** **and** **b)**, we defined cases as drug use alone, and drug use or diagnosis of hypertension. For primary care data (FIRE) **(c** **and** **d)**, we defined cases as drug use alone, drug use or diagnosis of hypertension, drug use or diagnosis of hypertension or measurement of increased blood pressure. For hospital discharge statistics (MEDSTAT) **(e** **and** **f)**, we defined cases as ICD-10 codes I10-I15 or I67.4 as secondary diagnoses.

SHS: The standardized prevalence estimates of hypertension defined as self-reported drug use alone, and self-reported drug use or self-reported diagnosis of hypertension, were 11.1% (95% CI 10.5-11.7%) and 19.2% (95% CI 18.3-20.1%), and 12.9% (95% CI 12.2-13.6%) and 23.7% (95% CI 22.7-24.8%) for women and men, respectively. The age- and gender-specific prevalence rates of hypertension for the different case definitions are provided in Figure 2a and b.FIRE: The standardized prevalence estimates of hypertension defined as drug prescription alone, drug prescription or diagnosis of hypertension, drug prescription or diagnosis of hypertension or measurement of high blood pressure, were 11.3% (95% CI 11.1-11.6%), 12.6% (95% CI 12.3-12.8%), 13.3% (95% CI 13.0-13.5%), and 14.2% (95% CI 14.0-14.5%), 15.8% (95% CI 15.5-16.1%), and 16.6% (95% CI 16.3-16.8%) for women and men, respectively. The age- and gender-specific prevalence rates of hypertension for the different case definitions are provided in Figure 2c and d.MEDSTAT: The standardized prevalence estimates of hypertension defined as ICD-10 codes I10-I15 as secondary diagnoses, were 10.8% (95% CI 10.7-10.8%) and 13.4% (95% CI 13.3-13.5%) for women and men, respectively. The age- and gender-specific prevalence rates of hypertension defined as ICD-10 codes E10-14 as secondary diagnoses are provided in Figure 2e and f.

### Dyslipidemia

**Figure 3:**
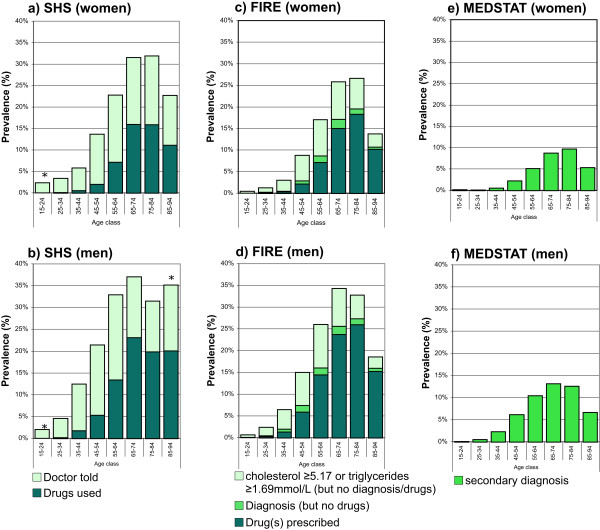
**Age- and gender-specific prevalence estimates of dyslipidemia based on different data sources and various definitions.** For health survey (SHS) **(a** **and** **b)**, we defined dyslipidemia as drug use alone, and drug use or diagnosis of dyslipidemia. For primary care data (FIRE) **(c** **and** **d)**, we defined dyslipidemia as drug use alone, drug use or diagnosis of lipid disorder, and drug use or diagnosis of lipid disorder or lipid measurements (either total cholesterol ≥5.17 mmol/L or triglycerides ≥1.69 mmol/L). For hospital discharge statistics (MEDSTAT) **(e** **and** **f)**, we defined dyslipidemia as ICD-10 code E78 as secondary diagnosis. Results based on less than 30 observations are marked by an asterisk.

SHS: The standardized prevalence estimates of dyslipidemia defined as self-reported drug use alone, and self-reported drug use or self-reported diagnosis of dyslipidemia were 3.7% (95% CI 3.3-4.1%) and 12.2% (95% CI 11.5-12.9%), and 6.3% (95% CI 5.7-6.8%) and 16.9% (95% CI 16.0-17.8%) for women and men, respectively. The age- and gender-specific prevalence rates of dyslipidemia for the two different case definitions are provided in Figure 3a and b.FIRE: The standardized prevalence estimates of dyslipidemia defined as drug prescription alone, drug prescription or diagnosis of lipid disorder, and drug prescription or diagnosis of lipid disorder or dyslipidemia based on measurement (either total cholesterol ≥5.17 mmol/L or triglycerides ≥1.69 mmol/L) were 3.7% (95% CI 3.6-3.9%), 4.4% (95% CI 4.2-4.6%), 8.5% (95% CI 8.3-8.7%), and 6.7% (95% CI 6.5-6.9%), 7.6% (95% CI 7.4-7.8%) and 12.7% (95% CI 12.4-12.9%) for women and men, respectively. The age- and gender-specific prevalence rates of dyslipidemia for the three different case definitions are provided in Figure 3c and d.MEDSTAT: The standardized prevalence estimates of dyslipidemia defined as ICD-10 code E78 as secondary diagnoses, were 2.5% (95% CI 2.4-2.5%) and 4.8% (95% CI 4.8-4.9%) for women and men, respectively. The age- and gender-specific prevalence rates of dyslipidemia defined as ICD-10 code E78 as secondary diagnoses are provided in Figure 3e and f.

### Obesity

**Figure 4:**
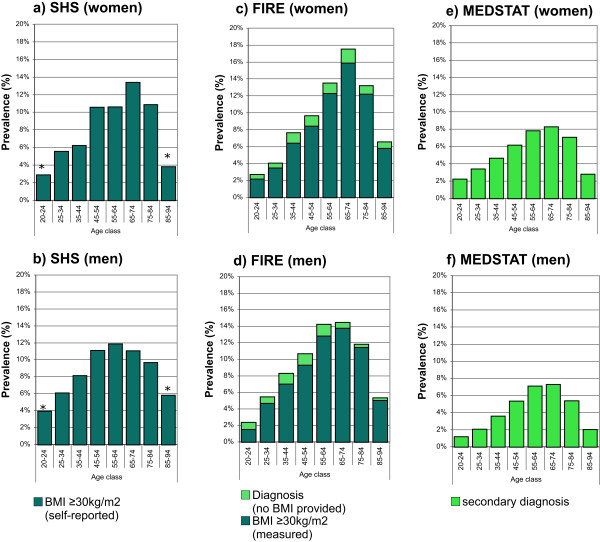
**Age- and gender-specific prevalence estimates of obesity based on complementary data sources and various definitions.** For health survey (SHS) **(a** **and** **b)**, we defined obesity as BMI ≥30 kg/m2 derived from self-reported height and weight. For primary care data (FIRE) **(c** **and** **d)**, we defined obesity as BMI ≥30 kg/m2 derived from measured height and weight, and BMI ≥30 kg/m2 derived from measured height and weight or diagnosis of obesity. For hospital discharge statistics (MEDSTAT) **(e** **and** **f)**, we defined obesity as ICD-10 code E66 as secondary diagnosis. Results based on less than 30 observations are marked by an asterisk.

SHS: The standardized prevalence estimates of obesity defined as BMI ≥30 kg/m2 derived from self-reported height and weight were 7.7% (95% CI 7.0-8.4%) and 8.4% (95% CI 7.5-9.2%) for women and men, respectively. The age- and gender-specific prevalence rates of obesity are provided in Figure 4a and b.FIRE: The standardized prevalence estimates of obesity defined as BMI ≥30 kg/m2 derived from measured height and weight, and BMI ≥30 kg/m2 derived from measured height and weight or diagnosis of obesity, were 7.4% (95% CI 7.0-7.8%) and 8.4% (95% CI 8.0-8.8%), and 7.6% (95% CI 7.2-7.8%) and 8.6% (95% CI 8.2-9.0%) for women and men, respectively. The age- and gender-specific prevalence rates of obesity for the two different case definitions are provided in Figure 4c and d.MEDSTAT: The standardized prevalence rates of obesity defined as ICD-10 code E66 as secondary diagnosis were 4.5% (95% CI 4.5-4.6%) and 3.9% (95% CI 3.8-4.0%) for women and men, respectively. The age-and gender-specific prevalence rates of obesity defined as ICD-10 code E66 as secondary diagnosis are provided in Figure 4e and f.

### Comparison

#### Diabetes mellitus

**Figure 5:**
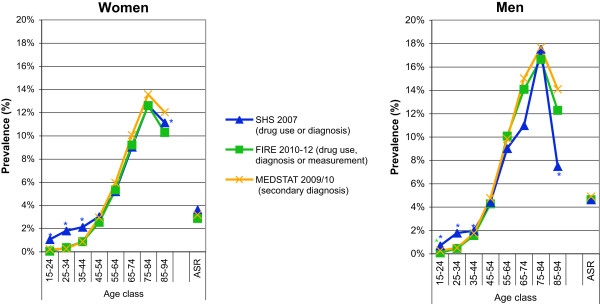
**Comparison of age- and gender-specific prevalence estimates of diabetes mellitus based on complementary data sources.** Diabetes mellitus was defined as drug use or diagnosis of diabetes mellitus in Swiss health survey (SHS), as drug use or diagnosis of diabetes mellitus or glucose measurement (serum glucose ≥11.1 mmol/L or HbA1c ≥6.5%) in primary care data (FIRE), and as ICD-10 codes E10-14 as secondary diagnoses in hospital discharge statistics (MEDSTAT). Results based on less than 30 observations are marked by an asterisk. (Data sources: Swiss Federal Statistical Office for Swiss Health Survey [SHS] and Hospital Discharge Statistics [MEDSTAT]; Swiss Family Medicine International Classification of Primary Care Research using Electronic Medical Record project for primary care data [FIRE]).

The best harmonization for the prevalence estimates of diabetes mellitus for the three different data sources was achieved using the most comprehensive definitions. The age- and gender-specific prevalence estimates of diabetes mellitus based on SHS (defined as drug use or diagnosis of diabetes mellitus), FIRE (defined as drug use or diagnosis or measurement [serum glucose ≥11.1 mmol/L or HbA1c ≥6.5%]), and MEDSTAT (defined as ICD-10 codes E10-14 as secondary diagnoses) are contrasted in Figure 5. These prevalence estimates were very similar for all three data sources, rising from less than 1% in the youngest age class (15-24 years), to more than 12% and 16% for women and men aged 75-84 years. A sharp decline in the prevalence estimates was observed in the oldest age class (85-94 years) of both genders.

#### Hypertension

**Figure 6:**
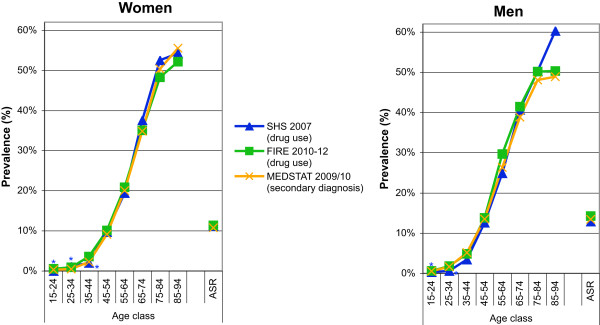
**Comparison of age- and gender-specific prevalence estimates of hypertension based on complementary data sources.** Hypertension was defined as drug use alone in Swiss Health Survey (SHS), as drug use alone in primary care data (FIRE), and as ICD-10 codes I10-I15 or I67.4 as secondary diagnoses in hospital discharge statistics (MEDSTAT). Results based on less than 30 observations are marked by an asterisk. (Data sources: Swiss Federal Statistical Office for Swiss Health Survey [SHS] and Hospital Discharge Statistics [MEDSTAT]; Swiss Family Medicine International Classification of Primary Care Research using Electronic Medical Record project for primary care data [FIRE]).

The best harmonization for the prevalence estimates of hypertension was achieved based on SHS (defined as drug use alone), FIRE (defined as drug use alone), and MEDSTAT (defined as ICD-10 codes I10-I15 as secondary diagnoses). The age- and gender-specific prevalence estimates (Figure 6) were very similar for the different data sources, increasing from less than 1% in the youngest age class (15-24 years), to more than 50% in the oldest age class (85-94 years).

#### Dyslipidemia

**Figure 7:**
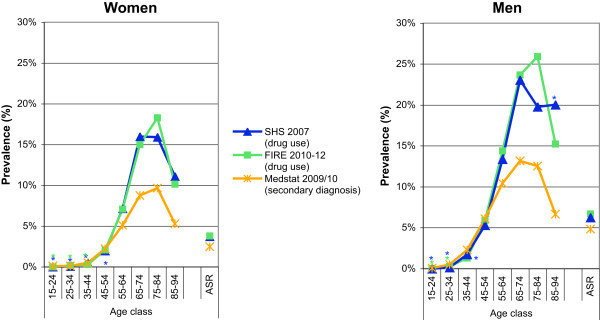
**Comparison of age- and gender-specific prevalence estimates of dyslipidemia based on complementary data sources.** Dyslipidemia was defined as drug use alone in Swiss Health Survey (SHS), as drug use alone in primary care data (FIRE), and as ICD-10 code E78 as secondary diagnosis in hospital discharge statistics (MEDSTAT). Results based on less than 30 observations are marked by an asterisk. (Data sources: Swiss Federal Statistical Office for Swiss Health Survey [SHS] and Hospital Discharge Statistics [MEDSTAT]; Swiss Family Medicine International Classification of Primary Care Research using Electronic Medical Record project for primary care data [FIRE]).

The best harmonization for the prevalence estimates of dyslipidemia were achieved based on SHS (defined as drug use alone), FIRE (defined as drug use alone), and MEDSTAT (defined as ICD-10 codes E78 as secondary diagnoses). The age- and gender-specific prevalence estimates are contrasted in Figure 7. There was a fair agreement between SHS and FIRE, with SHS underestimating slightly in the age class 75-84 years. However, MEDSTAT underestimated the prevalence rate systematically by close to 50% for all age groups, although the age- and gender-specific distribution remained similar. A sharp decline in prevalence of dyslipidemia was observed for the oldest age class (85-94 years) and for both genders in all three data sources.

#### Obesity

**Figure 8:**
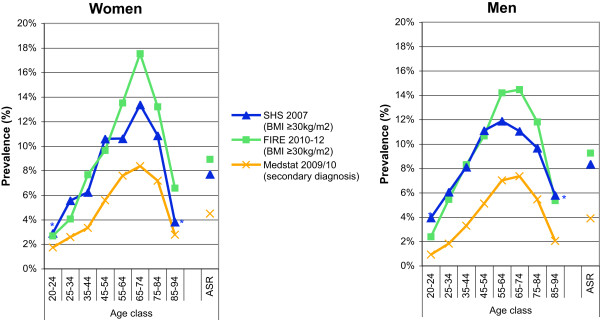
**Comparison of age- and gender-specific prevalence estimates of obesity based on complementary data sources.** Obesity was defined as BMI ≥30 kg/m2 derived from self-reported height and weight in Swiss Health Survey (SHS), as BMI ≥30 kg/m2 derived from measured height and weight or diagnosis of obesity in primary care data (FIRE), and as ICD-10 code s E66 as secondary diagnosis in hospital discharge statistics (MEDSTAT). Results based on less than 30 observations are marked by an asterisk. (Data sources: Swiss Federal Statistical Office for Swiss Health Survey [SHS] and Hospital Discharge Statistics [MEDSTAT]; Swiss Family Medicine International Classification of Primary Care Research using Electronic Medical Record project for primary care data [FIRE]).

The age- and gender-specific prevalence rates of obesity based on SHS (defined as BMI ≥30 kg/m2 derived from self-reported height and weight), FIRE (defined as BMI ≥30 kg/m2 derived from measured height and weight or diagnosis of obesity), and MEDSTAT (defined as ICD-10 code E66 as secondary diagnoses) are contrasted in Figure 8. Although there was generally a slight underestimation in SHS and severe underestimation in MEDSTAT, the age- and gender-specific prevalence distribution was similar for all data sources. A sharp decline in the prevalence of obesity was observed for the oldest age classes (75-84 and 85-94 years) and both genders in all data sources.

## Discussion

To date, only a few papers reported prevalence estimates derived from different data sources, usually contrasting national or regional health surveys with pooled data from primary care. Examples are studies from Spain [8, 9], the UK [10], Germany [11], and Italy [12]. All these studies aimed at scrutinizing the comparability of data from different sources in order to endorse the validity of prevalence estimates.

In our study, although the prevalence estimates varied substantially, depending on the methodology used to define a specific chronic medical condition (case definition) and on the nature of data source explored (health survey, primary care, and hospital discharge), a high degree of concordance could be achieved regarding age- and gender-related patterns for four common cardiovascular risk factors. Therefore, prevalence estimates of chronic medical conditions based on primary care data and hospital discharge statistics usually can be generalized to the population after a careful and appropriate harmonization of the case definitions.

### Comparison with other studies

In line with others [8–10, 13], concordance between health survey and primary care data was good for clearly defined chronic conditions (diabetes and hypertension), and fair for chronic conditions with a less clear cut-off (dyslipidemia and obesity). While for diabetes comprehensive definitions performed best, for hypertension and dyslipidemia building the case definition on drug use alone was more efficient. Hospital data performed well for clearly defined diagnoses (diabetes, hypertension), but underreported severely chronic conditions with a less clear cut-off (dyslipidemia, obesity).

Our prevalence estimates are fairly in accordance with the few Swiss studies, which explored the prevalence rates of cardiovascular risk factors. However, generalizability of these studies cannot be taken for granted, since they were either limited to a single city [14–16] or based on pharmacy claims with no access to clinical information [17] and probably limited regarding international comparability [18].

### Strengths and limitations

The main strength of our study is the inclusion of three and not only two data sources, which provide complementary information and open possibilities for further analyses of other chronic conditions. While there have been efforts to compare health survey and primary care data in several countries – to our knowledge the inclusion of hospital discharge statistics is rather novel.

Limitations of our study are due to properties of the different data sources. For instance, the SHS has a rather small number of participants and is restricted to self-reports. Participation is voluntary and therefore incomplete (66%), promoting a selection bias toward health-aware individuals, potentially having less chronic conditions but also favoring over reporting. FIRE on the other side, has a limited number of participating practices (81), which are not necessarily representative of the study region. Furthermore, FIRE captures the actual prevalence of chronic conditions among persons attending general practices, and not necessarily the prevalence in the general population, thus favoring over estimation due to selection bias of sicker patients. FIRE is based on voluntary participation and might have incomplete data. Similarly, MEDSTAT is limited to inpatients, which might have more chronic medical conditions than the general population. These characteristics have to be taken into consideration when drawing conclusions about the general population. Nevertheless, SHS, FIRE and MEDSTAT represent currently the largest and most comprehensive health data sources in Switzerland.

One could argue that comparisons between the data sources are problematic due to different definitions of the chronic conditions explored. However, our results show that a careful harmonization of the definitions is the key method. Since our study focused on the complementation of different data sources, an important question concerning definitions of chronic conditions remained unanswered: How arbitrary are the definitions of chronic health conditions based on cut-off-values, for instance dyslipidemia? Both, the definition based on lipid values and the definition based on drug use are fallible, because the lipid cut-off values are based on a variable expert opinion and the indication for medication depends on the patients’ need for secondary prevention (while drug use for primary prevention remains disputed). As a result, none of the definitions is comprehensive and all are prone to over- or underestimating bias. The identification of hypertension based on drug use is even more complicated because antihypertensive drugs can be used for heart disease or renal disease as well. However, many patients with renal disease or heart disease have hypertension. We calculated the prevalence rates for hypertension including and excluding patients who had renal disease or heart disease, and found minimal differences (not shown). Therefore, we provide prevalence estimates for hypertension based on antihypertensive drug use without excluding patients with heart failure or renal disease.

## Conclusion

We conclude that complementary data from different data sources might generate different prevalence estimates of chronic medical conditions in the general population. However, common age and sex patterns indicate that a careful harmonization of the definitions of chronic health conditions will provide strikingly similar age-and gender-specific distributions, even in data sources based on different settings and assessment methods. This kind of cross-validation opens vast potentials for analyses of prevalence estimates of other chronic health conditions as well as multimorbidity in specific subpopulations. Still, the development of generally accepted condition-specific case definitions remains crucial.
